# Supplementary material for: Integrating muti-omics data to identify tissue-specific DNA methylation biomarkers for cancer risk
Source: Nat Commun. 2024 Jul 18;15:6071. doi: 10.1038/s41467-024-50404-y (PMC11258330; doi:10.1038/s41467-024-50404-y)
Supplement: Supplementary file 1 — Supplementary Information [file 41467_2024_50404_MOESM1_ESM.pdf]

Integrating muti-omics data to identify tissue-specific DNA methylation biomarkers for cancer risk

Yaohua Yang[1†*], Yaxin Chen[2†], Shuai Xu[3], Xingyi Guo[3], Guochong Jia[3], Jie Ping[3], Xiang Shu[4], Tianying Zhao[3], Fangcheng Yuan[3], Gang Wang[2], Yufang Xie[2], Hang Ci[2], Hongmo Liu[2], Yawen Qi[2], Yongjun Liu[5], Dan Liu[2], Weimin Li[2], Fei Ye[6], Xiao-Ou Shu[3], Wei Zheng[3], Li Li[7], Qiuyin Cai[3*], Jirong Long[3*]

* Corresponding authors contact information:

Dr. Yaohua Yang, Center for Public Health Genomics, School of Medicine, University of Virginia. Address: 560 Ray C. Hunt Dr, Rm 4408, Charlottesville, VA 22903. Email: vta8we@virginia.edu

Dr. Qiuyin Cai, Division of Epidemiology, Department of Medicine, Vanderbilt University Medical Center. Address: 1161 21st Avenue South, MCN B-2104, Nashville, TN, 37232. Email: qiuyin.cai@vanderbilt.edu

Dr. Jirong Long, Division of Epidemiology, Department of Medicine, Vanderbilt University Medical Center. Address: 2525 West End Avenue, Suite 800, Nashville, TN, 37203. Email: jirong.long@vumc.org

† These authors contribute equally to this work

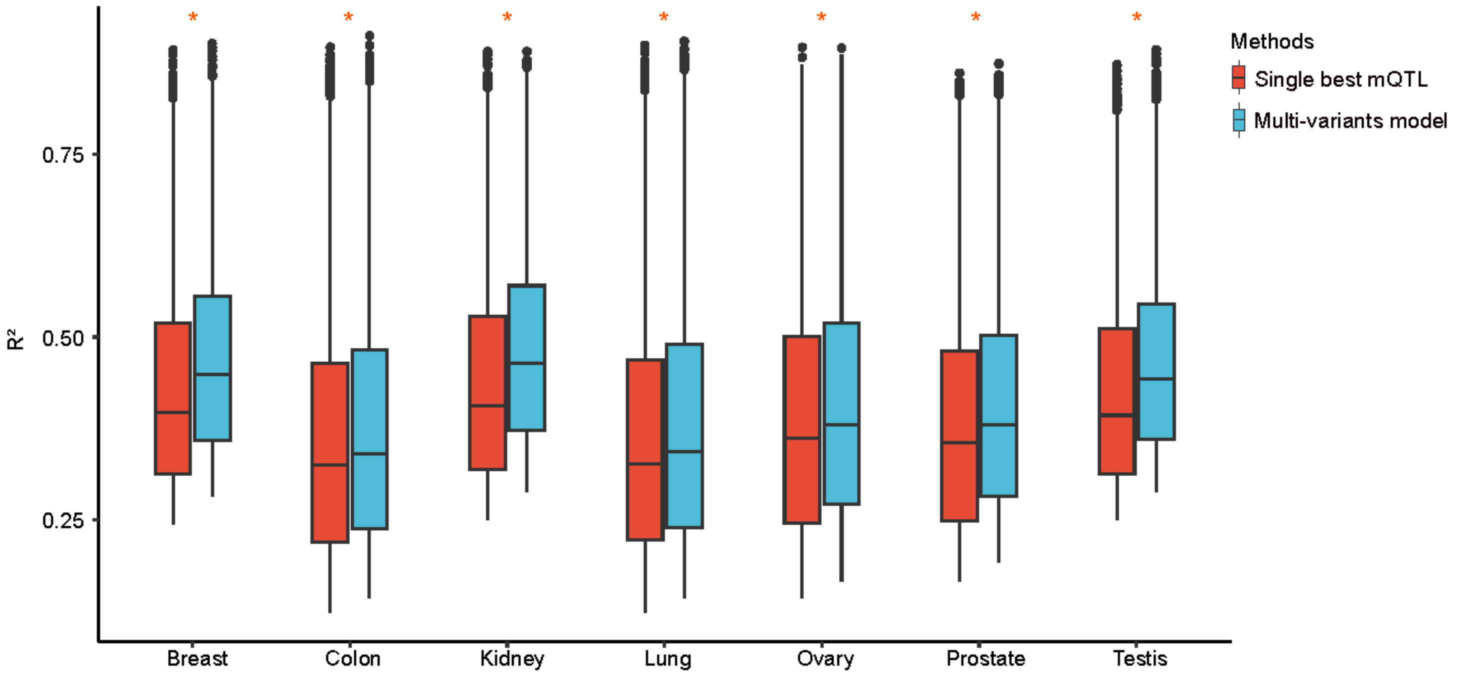

Supplementary Fig. 1. Prediction performance of models established by the present study and mQTLs. CpGs that could be reliably predicted (R>0.10 and *P*<0.05) by both our prediction models and mQTLs were used for comparison. The number of such CpGs is n=24,604 for breast tissue, n=85,285 for colon tissue, n=31,296 for kidney tissue, n=87,987 for lung tissue, n=76,161 for ovary tissue, n=51,193 for prostate tissue, and n=24,297 for testis tissue. Boxes represent the interquartile range, black bars are medians, whiskers extend at most 1.5 times the interquartile range, and outliers are shown individually. Red asterisk denotes *P*≈0 in two-sided paired t-test. mQTL, methylation quantitative trait loci. Source data are provided as a Source Data file.

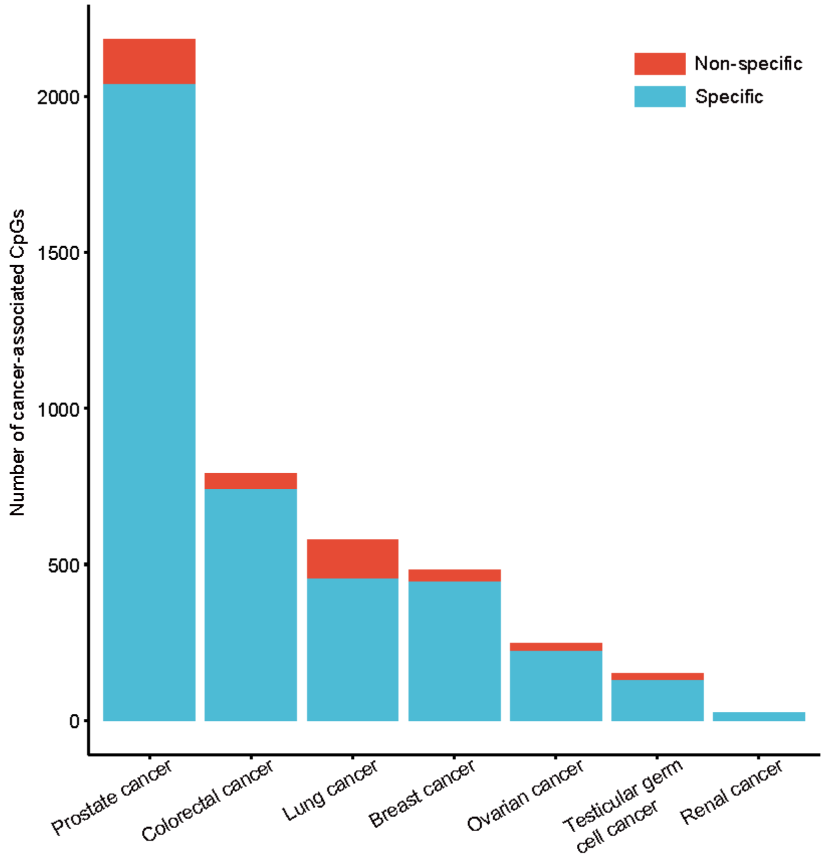

Supplementary Fig. 2. **Tissue-specificity of cancer-associated CpGs**. For each cancer, association analyses of genetically predicted DNA methylation (DNAm) with cancer risk were performed using SPrediXcan and significant associations were identified at Bonferroni-corrected two-side P<0.05, corresponding to 4.93×10^{-7} for breast cancer (n=101,497 CpGs tested; n=539,198 subjects), 2.53×10^{-7} for colorectal cancer (n=197,947 CpGs tested; n=254,791 subjects), 3.98×10^{-7} for renal cell cancer (n=125,745 CpGs tested; n=31,190 subjects), 2.55×10^{-7} for lung cancer (n=195,764 CpGs tested; n=887,170 subjects), 2.66×10^{-7} for ovarian cancer (n=187,911 CpGs tested; n=70,668 subjects), 3.28×10^{-7} for prostate cancer (n=152,341 CpGs tested; n=944,762 subjects), and 4.22×10^{-7} for testicular germ cell cancer (n=118,568 CpGs tested; n=28,135 subjects). CpGs that were exclusively associated with a particular cancer at Bonferroni-corrected P<0.05 are considered tissue specific. Source data are provided as a Source Data file.

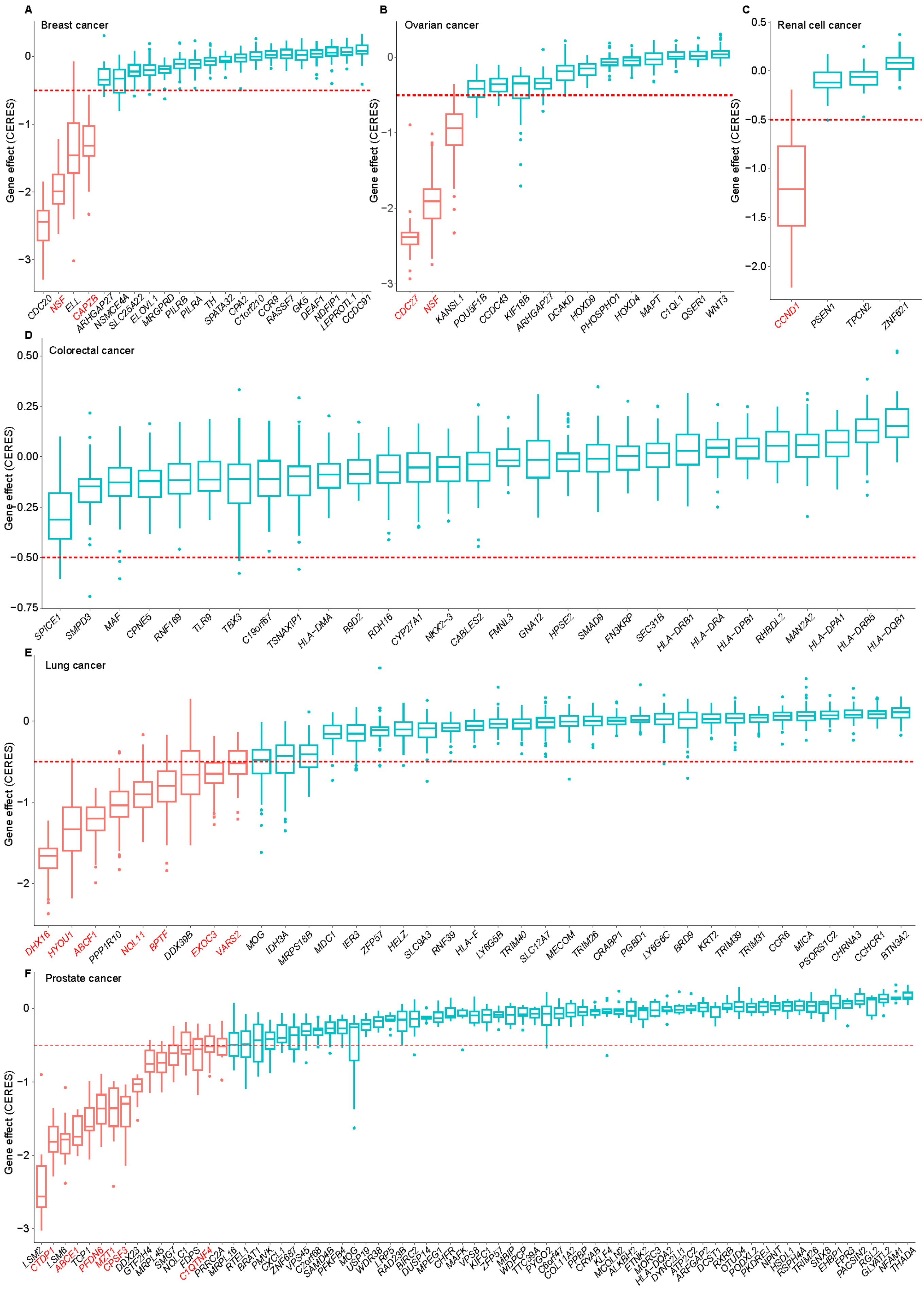

A
Breast cancer
Gene effect (CERES)
0
−1
−2
−3
CDC20
NSF
ELL
CAPZB
ARHGAP27
NSMCE4A
SLC25A22
ELOVL1
MRGPRD
PILRB
PILRA
TH
SPATA32
CPA2
C1orf210
CCR9
RASSF7
GK5
DEAF1
NDFIP1
LEPROTL1
CCDC91
B
Ovarian cancer
Gene effect (CERES)
CDC27
NSF
KANSL1
POU5F1B
CCDC43
KIF18B
ARHGAP27
DCAKD
HOXD9
PHOSPHO1
HOXD4
MAPT
C1QL1
QSER1
WNT3
C
Renal cell cancer
Gene effect (CERES)
0.5
0.0
−0.5
−1.0
−1.5
−2.0
CCND1
PSEN1
TPCN2
ZNF621
D
Colorectal cancer
Gene effect (CERES)
0.50
0.25
0.00
−0.25
−0.50
−0.75
SPICE1
SMPD3
MAF
CPNE5
RNF169
TLR9
TBX3
C19orf67
TSNAXIP1
HLA−DMA
B9D2
RDH16
CYP27A1
NKX2−3
CABLES2
FMNL3
GNA12
HPSE2
SMAD9
FN3KRP
SEC31B
HLA−DRB1
HLA−DRA
HLA−DPB1
RHBDL2
MAN2A2
HLA−DPA1
HLA−DRB5
HLA−DQB1
E
Lung cancer
Gene effect (CERES)
DHX16
HYOU1
ABCF1
PPP1R10
NOL11
BPTF
DDX39B
EXOC3
VARS2
MOG
IDH3A
MRPS18B
MDC1
IER3
ZFP57
HELZ
SLC9A3
RNF39
HLA−F
LY6G5B
TRIM40
SLC12A7
MECOM
TRIM26
CRABP1
PGBD1
LY6G6C
BRD9
KRT2
TRIM39
TRIM31
CCR6
MICA
PSORS1C2
CHRNA3
CCHCR1
BTN3A2
F
Prostate cancer
Gene effect (CERES)
LSM2
CTDP1
LSM6
ABCE1
TCP1
PFDN6
MZT1
CPSF3
DDX23
GTF2H4
MRPL45
SMG7
NOLC1
FDPS
C1QTNF4
PRRC2A
MRPL16
RTEL1
BRAT1
PMVK
CXCL1
ZNF687
VPS45
C2orf68
SAMD4B
PFKFB4
MOG
USP19
WDR38
LRP5
RAD23B
BIRC2
DUSP14
MPEG1
CHFR
MAFK
VPS8
KIFC1
ZFP57
MBIP
WDPCP
TTC39A
PYGO2
C8orf47
COL11A2
PPBP
CRYAB
KLF4
MCOLN2
ALKBH2
ETNK2
MORC3
HLA−DQA2
DYNC2LI1
ATP2C2
ARFGAP2
DCST1
RXRB
OTUD4
PODXL2
PKDREJ
NPNT
HSDL1
RSPH4A
TRIM26
SNX8
EHBP1
FPR3
PACSIN2
RGL2
GLYATL2
NFAM1
THADA

Supplementary Fig. 3. Essential roles in cell proliferation of genes associated with cancer-associated CpGs. Boxplots show effects of genes on cell proliferation using experimental data from Clustered Regularly Interspaced Short Palindromic Repeats (CRISPR) screens. Boxes represent the interquartile range, black bars are medians, whiskers extend at most 1.5 times the interquartile range, and outliers are shown individually. The dashed red line in each plot denotes the essentiality threshold of median CERES values<-0.50. Genes shown in CpG-gene-cancer trios revealed by downstream analyses (see **Methods**) are highlighted in red. **A**, breast cancer (n=48 cell lines). **B**, ovarian cancer (n=57 cell lines). **C**, renal cell cancer (n=32 cell lines). **D**, colorectal cancer (n=57 cell lines). **E**, lung cancer (n=114 cell lines). **F**, prostate cancer (n=10 cell lines). Source data are provided as a Source Data file.

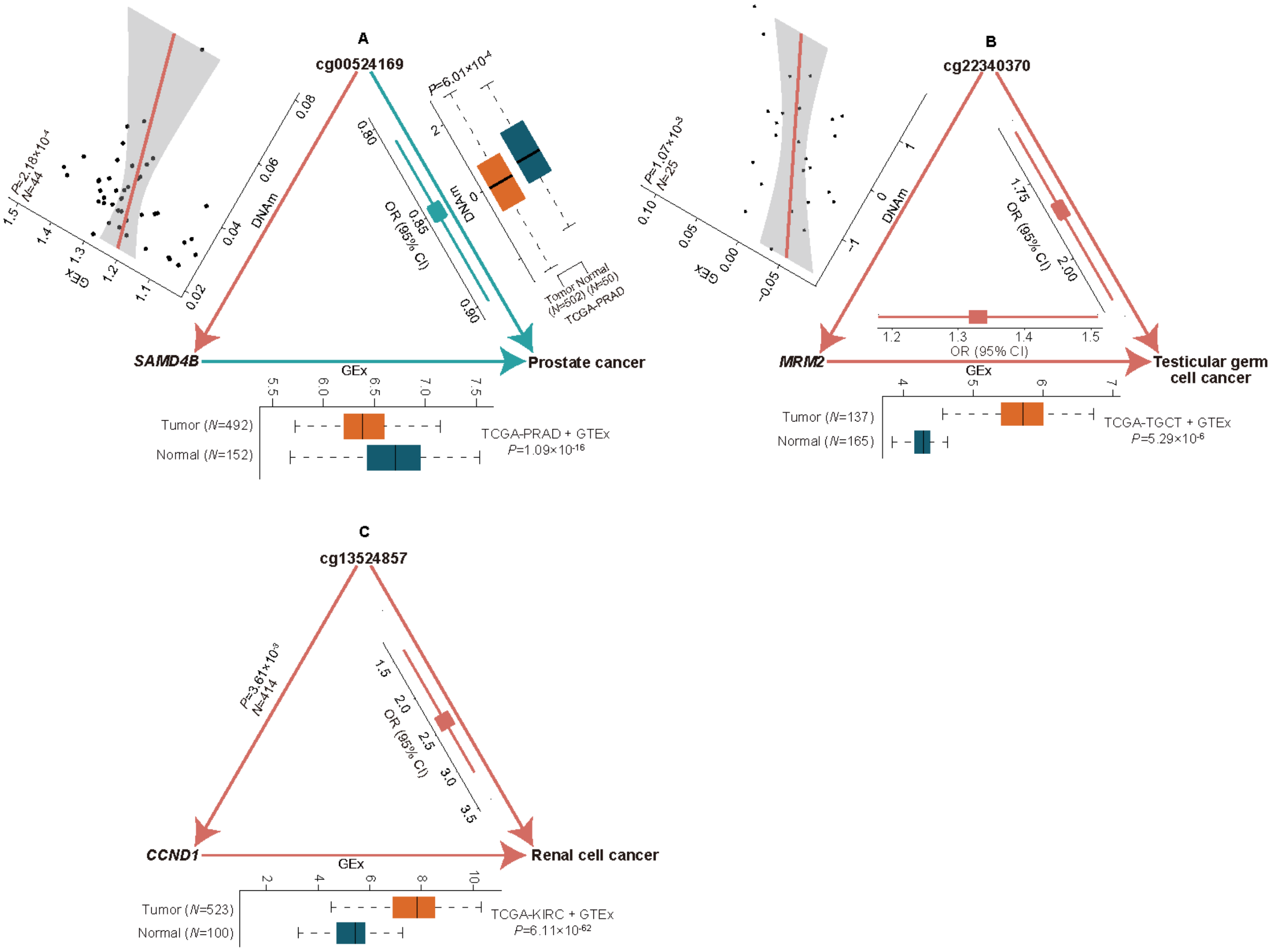

Supplementary Fig. 4. **Additional examples of CpG-gene-cancer trios suggesting DNA methylation influencing cancer risk by modulating *cis*-gene expression**. Association analyses of genetically predicted DNA methylation (DNAm) or gene expression (GEx) with cancer risk were performed using SPrediXcan. Differential DNAm or GEx analyses were conducted using linear mixed models. Association analyses between DNAm and GEx were performed using linear regression. Red arrows, lines, and blocks denote positive associations, while green ones denote negative associations. All statistical tests were two-sided and multiple comparisons were Bonferroni- or false discovery rate (FDR)-adjusted. The odds ratio (OR) and 95% confidence interval (CI) for cancer risk per standard deviation (SD) increase in genetically predicted DNAm or GEx are displayed as a block with error bands. In

boxplots, boxes represent the interquartile range, black bars are medians, and whiskers extend at most 1.5 times the interquartile range. In the scatter plot displaying the association between DNAm and GEx, directly measured DNAm and GEx values after quantile- and inverse-normalization are presented. n, number of samples, TCGA, The Cancer Genome Atlas; GTEx, Gene-Tissue Expression consortium; PRAD, prostate adenocarcinoma; TGCT, testicular germ cell tumors; KIRC, kidney renal clear cell carcinoma. **A**, DNAm at cg00524169 may decrease prostate cancer risk by promoting the expression of *SAMD4B*. The sample size was 552 for tumor-normal differential DNAm analyses, 44 for DNAm-GEx correlation analyses, 644 for tumor-normal differential GEx analyses, and 944,762 for association analyses of predicted DNAm and GEx with prostate cancer risk, respectively. **B**, DNAm at cg22340370 may elevate testicular germ cell cancer risk by promoting the expression of *MRM2*. The sample size was 25 for DNAm-GEx correlation analyses, 302 for tumor-normal differential GEx analyses, and 28,135 for association analyses of predicted DNAm and GEx with testicular germ cell cancer risk, respectively. **C**, DNAm at cg13524857 may increase renal cell cancer risk by promoting the expression of *CCND1*. The sample size was 414 for DNAm-GEx correlation analyses, 623 for tumor-normal differential GEx analyses, and 31,190 for association analyses of predicted DNAm and GEx with renal cell cancer risk, respectively. Source data are provided as a Source Data file.
