# Supplementary material for: Integrating muti-omics data to identify tissue-specific DNA methylation biomarkers for cancer risk
Source: Nat Commun. 2024 Jul 18;15:6071. doi: 10.1038/s41467-024-50404-y (PMC11258330; doi:10.1038/s41467-024-50404-y)
Supplement: Supplementary file 3 — Description of Additional Supplementary Files [file 41467_2024_50404_MOESM3_ESM.pdf]

Description of Additional Supplementary Files

File Name: Supplementary Data 1
Description: Information on summary statistics data from genome-wide association studies of cancers.

File Name: Supplementary Data 2
Description: CpGs significantly associated with breast cancer risk.

File Name: Supplementary Data 3
Description: CpGs significantly associated with colorectal cancer risk.

File Name: Supplementary Data 4
Description: CpGs significantly associated with renal cell cancer risk.

File Name: Supplementary Data 5
Description: CpGs significantly associated with lung cancer risk.

File Name: Supplementary Data 6
Description: CpGs significantly associated with ovarian cancer risk.

File Name: Supplementary Data 7
Description: CpGs significantly associated with prostate cancer risk.

File Name: Supplementary Data 8
Description: CpGs significantly associated with testicular germ cell cancer risk.

File Name: Supplementary Data 9
Description: Distributions of breast cancer-associated CpGs, genes and splicing variants within breast cancer susceptibility loci identified by previous GWAS.

File Name: Supplementary Data 10
Description: Distributions of colorectal cancer-associated CpGs, genes and splicing variants within colorectal cancer susceptibility loci identified by previous GWAS.

File Name: Supplementary Data 11
Description: Distributions of renal cell cancer-associated CpGs and genes within renal cell cancer susceptibility loci identified by previous GWAS.

File Name: Supplementary Data 12
Description: Distributions of lung cancer-associated CpGs, genes and splicing variants within lung cancer susceptibility loci identified by previous GWAS.

File Name: Supplementary Data 13
Description: Distributions of ovarian cancer-associated CpGs, genes and splicing variants within ovarian cancer susceptibility loci identified by previous GWAS.

File Name: Supplementary Data 14

Description: Distributions of prostate cancer-associated CpGs, genes and splicing variants within prostate cancer susceptibility loci identified by previous GWAS.

File Name: Supplementary Data 15
Description: Distributions of testicular germ cell cancer-associated CpGs, genes and splicing variants within testicular germ cell cancer susceptibility loci identified by previous GWAS.

File Name: Supplementary Data 16
Description: Breast cancer-associated CpGs showing consistent differential DNA methylation between breast cancer and adjacent normal tissues in TCGA.

File Name: Supplementary Data 17
Description: Colorectal cancer-associated CpGs showing consistent differential DNA methylation between colorectal adenocarcinoma and adjacent normal tissues in TCGA.

File Name: Supplementary Data 18
Description: Renal cell cancer-associated CpGs showing consistent differential DNA methylation between renal cell cancer and adjacent normal tissues in TCGA.

File Name: Supplementary Data 19
Description: Lung cancer-associated CpGs showing consistent differential DNA methylation between lung cancer and adjacent normal tissues in TCGA.

File Name: Supplementary Data 20
Description: Prostate cancer-associated CpGs showing consistent differential DNA methylation between prostate cancer and adjacent normal tissues in TCGA.

File Name: Supplementary Data 21
Description: CpG-gene-cancer trios suggesting DNA methylation influencing breast cancer risk by regulating gene expression.

File Name: Supplementary Data 22
Description: CpG-gene-cancer trios suggesting DNA methylation influencing colorectal cancer risk by regulating gene expression.

File Name: Supplementary Data 23
Description: CpG-gene-cancer trios suggesting DNA methylation influencing renal cell cancer risk by regulating gene expression.

File Name: Supplementary Data 24
Description: CpG-gene-cancer trios suggesting DNA methylation influencing lung cancer risk by regulating gene expression.

File Name: Supplementary Data 25
Description: CpG-gene-cancer trios suggesting DNA methylation influencing ovarian cancer risk by regulating gene expression.

File Name: Supplementary Data 26
Description: CpG-gene-cancer trios suggesting DNA methylation influencing prostate cancer risk by regulating gene expression.

File Name: Supplementary Data 27
Description: CpG-gene-cancer trios suggesting DNA methylation influencing testicular germ cell cancer risk by regulating gene expression.
