# Supplementary material for: Integrating muti-omics data to identify tissue-specific DNA methylation biomarkers for cancer risk
Source: Nat Commun. 2024 Jul 18;15:6071. doi: 10.1038/s41467-024-50404-y (PMC11258330; doi:10.1038/s41467-024-50404-y)
Supplement: Supplementary file 5 — Reporting Summary [file 41467_2024_50404_MOESM5_ESM.pdf]

nature portfolio

Corresponding author(s): Yaohua Yang, Qiuyin Cai, Jirong Long

Last updated by author(s): Jun 25, 2024

Reporting Summary

Nature Portfolio wishes to improve the reproducibility of the work that we publish. This form provides structure for consistency and transparency in reporting. For further information on Nature Portfolio policies, see our Editorial Policies and the Editorial Policy Checklist.

Statistics

For all statistical analyses, confirm that the following items are present in the figure legend, table legend, main text, or Methods section.

n/a	Confirmed	
☐	☒	The exact sample size (*n*) for each experimental group/condition, given as a discrete number and unit of measurement
☒	☐	A statement on whether measurements were taken from distinct samples or whether the same sample was measured repeatedly
☐	☒	The statistical test(s) used AND whether they are one- or two-sided<br>*Only common tests should be described solely by name; describe more complex techniques in the Methods section.*
☐	☒	A description of all covariates tested
☐	☒	A description of any assumptions or corrections, such as tests of normality and adjustment for multiple comparisons
☐	☒	A full description of the statistical parameters including central tendency (e.g. means) or other basic estimates (e.g. regression coefficient) AND variation (e.g. standard deviation) or associated estimates of uncertainty (e.g. confidence intervals)
☐	☒	For null hypothesis testing, the test statistic (e.g. *F*, *t*, *r*) with confidence intervals, effect sizes, degrees of freedom and *P* value noted<br>*Give P values as exact values whenever suitable.*
☒	☐	For Bayesian analysis, information on the choice of priors and Markov chain Monte Carlo settings
☒	☐	For hierarchical and complex designs, identification of the appropriate level for tests and full reporting of outcomes
☒	☐	Estimates of effect sizes (e.g. Cohen's *d*, Pearson's *r*), indicating how they were calculated

Our web collection on statistics for biologists contains articles on many of the points above.

Software and code

Policy information about availability of computer code

Data collection	All data used in this study were manually collected from different sources, including NHGRI-EBI GWAS Catalogue, dbGaP, GDC Data Portal, GEPIA, GTEx portal, PredictDB.
Data analysis	All data used in this study were manually collected from different sources, including NHGRI-EBI GWAS Catalogue, dbGaP, GDC Data Portal, GEPIA, GTEx portal, PredictDB. The prediction models derived in this study, along with the code that was used to generated these models, are publicly available without any restrictions at https://zenodo.org/records/10810820. These information has been included in the "Data Availability" and "Code Availability" sections of the manuscript.<br><br>All analyses were performed using Python (v3.6.3) and/or R (v3.6.0). DNA methylation prediction model development was conducted using the R package glmnet (v4.1-8) and the UTMOST (2023 release) software. Association analyses of genetically predicted DNA methylation or gene expression with cancer risk were performed using SPrediXcan (v0.7.5). Differential DNA methylation or gene expression analyses were conducted using linear mixed models implemented in the R package nlme (v3.1.140). Association analyses between DNA methylation and gene expression were performed using linear regression using R.

For manuscripts utilizing custom algorithms or software that are central to the research but not yet described in published literature, software must be made available to editors and reviewers. We strongly encourage code deposition in a community repository (e.g. GitHub). See the Nature Portfolio guidelines for submitting code & software for further information.

Data

Policy information about availability of data

All manuscripts must include a data availability statement. This statement should provide the following information, where applicable:

- Accession codes, unique identifiers, or web links for publicly available datasets
- A description of any restrictions on data availability
- For clinical datasets or third party data, please ensure that the statement adheres to our policy

The publicly available genotype, DNA methylation, and gene expression data of GTEx participants used in this study are available in the dbGaP and GEO under accession code phs000424.v8.p2 [https://www.ncbi.nlm.nih.gov/projects/gap/cgi-bin/study.cgi?study_id=phs000424.v8.p2] and GSE213478 [https://www.ncbi.nlm.nih.gov/geo/query/acc.cgi?acc=GSE213478]. respectively.
The publicly available DNA methylation data of TCGA participants used in this study are available in the NCI Genomic Data Commons Data Portal [https://portal.gdc.cancer.gov/].
The publicly available GTEx v8-based gene expression and splicing prediction models used in this study are available in PredictDB [https://predictdb.org/].
The publicly available differential gene expression data used in this study are available in GEPIA2 [http://gepia2.cancer-pku.cn/#index].
The publicly available summary statistics of cancer GWAS used in this study are available in Zenodo (accession code 7814694 [https://zenodo.org/records/7814694#.ZDaspXbMK5d]) for breast cancer, GWAS catalogue (accession code GCST90129505 [https://www.ebi.ac.uk/gwas/studies/GCST90129505]) for colorectal cancer, dbGaP for renal cell cancer (accession code phs001736.v2.p1 [https://www.ncbi.nlm.nih.gov/projects/gap/cgi-bin/study.cgi?study_id=phs001736.v2.p1]), GWAS catalogue (accession code GCST004746 [https://www.ebi.ac.uk/gwas/studies/GCST004746], GCST90277434 [https://www.ebi.ac.uk/gwas/studies/GCST90277434]), UK Biobank data from the Neale lab (https://www.nealelab.is/uk-biobank), the FinnGen website (https://r9.finngen.fi/), and the Biobank Japan website (https://pheweb.jp/) for lung cancer, the OCAC website (https://ocac.ccge.medschl.cam.ac.uk/data-projects/) for ovarian cancer, GWAS catalogue (accession code GCST90274713 [https://www.ebi.ac.uk/gwas/studies/GCST90274713]) for prostate cancer, and dbGaP (accession code phs001349 [https://www.ncbi.nlm.nih.gov/projects/gap/cgi-bin/study.cgi?study_id=phs001349.v1.p1]) for testicular germ cell cancer.
The DNA methylation prediction models generated in this study have been deposited in Zenodo (accession code 10810820 [https://zenodo.org/records/10810820]). The remaining data are available within the Article, Supplementary Information or Source Data files.

Research involving human participants, their data, or biological material

Policy information about studies with human participants or human data. See also policy information about sex, gender (identity/presentation), and sexual orientation and race, ethnicity and racism.

Reporting on sex and gender	In all analyses, self-reported sex were reported and adjusted as a covariate. Except for sex-specific cancer types, analyses stratified by sex were not performed because of the unavailability of cancer GWAS data stratified by sex.
Reporting on race, ethnicity, or other socially relevant groupings	Our study included subjects of diverse ancestries. In DNA methylation prediction model development, we used data from individuals of European (N=318), African (N=44), Asian (N=4), and American Indian/Alaska Native (N=1). For cancer GWAS data, except for renal cell cancer and testicular germ cell cancer, for which data among non-European populations are unavailable, data for all the other five cancers were from multi-ancestry meta-analyses. Specifically, for colorectal and prostate cancers, we used to-date the largest cross-ancestry GWAS data from recently published studies, in which data for colorectal cancer included European- and Asian-ancestry subjects, while that for prostate cancer include European, African, Asian, and Hispanic/Latino subjects. For breast, ovarian, and lung cancers, we performed meta-analyses to combine data from different ancestral populations. We provided detailed information on these datasets in Supplementary Table S1.
Population characteristics	All GTEx participants included in DNA methylation prediction model development were cancer-free subjects, aged 20 to 70 years (median: 55, interquartile range 46-64). Characteristics of study participants included in cancer GWAS were described in detail in relevant publications of GWAS consortia and cited in our submitted manuscript.
Recruitment	This study used data from published studies. No new recruitment was performed.
Ethics oversight	All data used in this study have obtained ethnic approval from their parent studies.

Note that full information on the approval of the study protocol must also be provided in the manuscript.

Field-specific reporting

Please select the one below that is the best fit for your research. If you are not sure, read the appropriate sections before making your selection.

☒ Life sciences ☐ Behavioural & social sciences ☐ Ecological, evolutionary & environmental sciences

For a reference copy of the document with all sections, see nature.com/documents/nr-reporting-summary-flat.pdf

Life sciences study design

All studies must disclose on these points even when the disclosure is negative.

Sample size	All analyses in this work utilized publicly available data, therefore no statistical method was used to predetermine sample size.

All data used in our study are from previously published studies, protocols of which have been approved by their corresponding boards/ committees.

Detailed information on sample size of studies in this work is described below.

1. Sample sizes of tissue samples for DNA methylation prediction model development.
49 breast tissues, 189 colon tissues, 47 kidney tissues, 190 lung tissues, 140 ovary tissues, 105 prostate tissues, 47 testis tissues, 47 whole blood tissues, and 42 muscle tissues.

2. Sample sizes of cancer GWAS:
Breast cancer : 158,742 cases and 380,456 controls
Colorectal cancer: 100,204 CRC cases and 154,587 controls
Renal cell cancer: 10,784 cases and 20,406 controls
Lung cancer: 50,503 cases and 836,667 controls
Ovarian cancer: 25,644 cases and 45,024 controls
Prostate cancer: 156,319 cases and 788,443 controls
Testicular germ cell cancer: including 10,156 cases and 17,979 controls.

Data exclusions	For DNA methylation model development, we excluded 57 subjects lacking genetic data, along with DNA methylation data of the 131 samples donated by them. No data exclusion was performed for cancer GWAS data.
Replication	The codes that could be used to replicate all of our findings from this work have been deposited in Zenodo under accession code 10810820 [https://zenodo.org/records/10810820]. Using these code and data, all results presented in this study can be replicated.
Randomization	Randomization is not applicable because this study analyzed publicly available data.
Blinding	The investigators were not blinded to allocation during the experiments and outcome assessment, because the data are not from controlled randomized studies.

Reporting for specific materials, systems and methods

We require information from authors about some types of materials, experimental systems and methods used in many studies. Here, indicate whether each material, system or method listed is relevant to your study. If you are not sure if a list item applies to your research, read the appropriate section before selecting a response.

Materials & experimental systems

n/a	Involved in the study
☒	☐ Antibodies
☒	☐ Eukaryotic cell lines
☒	☐ Palaeontology and archaeology
☒	☐ Animals and other organisms
☒	☐ Clinical data
☒	☐ Dual use research of concern
☒	☐ Plants

Methods

n/a	Involved in the study
☒	☐ ChIP-seq
☒	☐ Flow cytometry
☒	☐ MRI-based neuroimaging

Plants

Seed stocks	Not applicable.
Novel plant genotypes	Not applicable.
Authentication	Not applicable.
